# Practical Hints

**Published:** 1877-03-20

**Authors:** 


### PRACTICAL HINTS.

The following formula is valuable in chronic affections of the skin—also a fine alterative in subacute or chronic rheumatism:

R. Iodide potassium..........dr. ij.
Fluid ext. stillingia........oz. j.
Fluid ext. polk root.......oz. ij.
Syrup.........................oz. j.
M.

Dose, teaspoonful three times a day.

### ANTIBILIOUS PURGATIVE POWDER.

The following preparation will be found very convenient and efficacious as an antibilious purgative to carry in your pocket-case, and not being disagreeable to the taste will be borne even by delicate stomachs. As it is very certain in its purgative action, there is little if any risk of salivation:

R. Calomel......................gr. x.
Podophylin...................gr. v.
Loaf sugar...............gr. xxx.
Bi carb. soda.............gr. xij.

Triturate and div. in powders No. xx. One powder will usually operate, producing bilious discharges. By practice the physician using this powder may learn to dose it correctly, on the point of his pocket-knife.

### INCREASE OF NERVOUS DISEASES.

Dr. Althans, in *Med. Times and Gaz.*, London, in answer to the question, "Do the conditions of modern life favor the development of nervous diseases, states in an elaborate statistical paper that such is not the case. We cannot answer for England, but feel well assured that, in this country, nervous affections, particularly paralyses and neuralgias, are far more frequent than they were twenty-five or thirty years ago. A fact which we think is in a large degree attributable to the use of tobacco, coffee, tea, and opium.

### BEST TIME TO DRESS FRACTURES.

Prof. Yandell, in a lecture to his class, answers the question as to the best time to dress a fracture, thus: "*The earliest possible moment after the bone is broken.*" This is common sense, and the idea that people, and often inexperienced doctors, have of removing the patient from the place of accident to his home, or other point, before dressing the fracture, is fraught with great risk and injury to fractured limbs. Dress it on the very spot, even if you have to go miles in search of material to do it with.

### SACCHARATED CALOMEL.

Calomel, when triturated thoroughly with loaf sugar, is greatly increased in power and efficiency by reason of the minute division of its molecules.

R. Calomel......................gr. x.
Loaf sugar. .. ... .........gr. xl. M.

Div. in powders No. xx.

One of these powders given every two to four hours will be found very efficacious in hepatic disorders.

### STRYCHNIA.

An excellent formula for administering:

R. Strychnia....................gr. j.
Acid acetic,
Tinc. cardamom com. aa dr. ss.
Spts. vin. rect.............oz. iss.
Syr. simp...................oz. iv. M.

Dose, teaspoonful three times per day.

### ERUPTIVE DISEASES OF THE SCALP.

The following has been found successful in many cases of the above affection:

R. Tannic acid................gr. xv.
Tinct. iodine... .........dr. ijss.
Glycerine...................dr. v.
Carbolic acid..............gtt. x. M.

Apply night and morning, first washing the part with water and castile soap.

